# Effects of Traceability Scope and Sample Quantity on Origin Tracing of Mineral Elements in Mung Beans

**DOI:** 10.1002/fsn3.71479

**Published:** 2026-01-28

**Authors:** Mingming Chen, Zhigang Quan, Lili Qian, Dongjie Zhang

**Affiliations:** ^1^ College of Food Science, Heilongjiang Bayi Agricultural University Daqing China; ^2^ Key Laboratory of Agri‐Products Processing and Quality Safety of Heilongjiang Province College of Food Science, Heilongjiang Bayi Agricultural University Daqing China; ^3^ National Coarse Cereals Engineering Research Center Daqing China

**Keywords:** accuracy, mineral element, mung bean, origin traceability, origin tracing model, sample quantity, traceability scope

## Abstract

Mineral element fingerprint analysis technology is one of the effective methods for grain origin identification. The accuracy of the identification model is closely related to sample origin, including traceability scope and sample quantity. In this study, mung bean samples from four sites (Tailai and Dorbod Mongol Autonomous County in Heilongjiang Province, Baicheng City in Jilin Province, and Sishui County in Shandong Province) were used as the research object. The contents of mineral elements in mung bean samples were determined by inductively coupled plasma mass spectrometry (ICP‐MS). Based on stoichiometric results, origin tracing models for different traceability scopes and different quantities of samples were established. The results show that the origin discrimination model established by the samples with different traceability scopes has the correct rate for the original discrimination of Tailai–Dorbod Mongol Autonomous (99.33%) < Tailai–Baicheng (99.67%) < Tailai–Sishui (100.0%). The origin discrimination model established by the different quantities has the correct rate for the original origin discrimination of 99.0% (*n* = 200) < 99.25% (*n* = 400) < 99.33% (*n* = 600). The results verified that the larger sample tracing scope and the larger sample quantity can improve the discrimination accuracy of the established origin tracing model.

## Graphical abstract

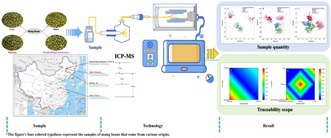

## 1 Introduction

The food geographical origin traceability and authenticity is very important for food safety surveillance, and it is also important for protecting sustainable development of geographical or specialty food industry. In the actual production and circulation of agricultural products, driven by economic interests, there is often food fraud. The occurrence of fraud, however, relates closely to asymmetric information (Ehmke et al. 2019), including incorrect identifications, with general food posing as famous and excellent and special products, cheating consumers. The mislabeling of agricultural and food products is one of the most common types of food fraud. Therefore, brand protection of Geographical Indications Products (CIP), protected designation of origin (PDO), protected geographical indication (PGI), and traditional speciality guaranteed (TSG) requires not only mandatory traceability together with a mandatory label (Cicia et al. 2005) but also increases the level of the current administrative fines (Charlebois et al. 2016). More urgent is the need to develop food authentication technology suitable for consumer use for the food marketplace (Bimbo et al. 2019).

The origin traceability technology of mineral elements is one of the effective methods to judge the authenticity of the origin traceability of agricultural products. It is mainly based on multi‐element analysis methods such as inductively coupled plasma mass spectrometry (ICP‐MS), inductively coupled plasma emission spectrometry (ICP‐ES), and has been widely explored in the origin traceability of rice (Maione et al. 2016; Chung et al. 2018), tea (Moreda‐Piñeiro et al. 2003; Zhao et al. 2017; Li et al. 2018), cowpea (Pérez‐Rodríguez et al. 2019), cocoa beans (Bertoldi et al. 2016), wolfberry (Zhang et al. 2017; Bertoldi et al. 2019), honey (Batista et al. 2012), wine (Korenovsk and Suhaj 2005; Coetzee et al. 2005; Mirabal Gallardo et al. 2018), virgin olive oil (Cabrera‐Vique et al. 2012; Beltrán et al. 2009), and other products. It is a common idea of agricultural product origin traceability technology by collecting samples of different geographical origins, based on inductively coupled plasma mass spectrometry analysis technology, and combining with stoichiometric methods, screening effective characteristic elements, and establishing the origin discrimination model. But the contents of mineral elements in agricultural products were related to the growing environment (soil (Richter et al. 2019), climate (Liu et al. 2019; Barbera et al. 2021), seasonality (Lima et al. 2021; Zhao and Yang 2019; Danezis and Georgiou 2022), and water (Liu et al. 2017)) and also affected by variety, plantation years, agricultural measures (chemical fertilizers (Song et al. 2021; Kratz et al. 2016), pesticides (Qian, Zhang, et al. 2019)), processing method (Qian, Zuo, et al. 2019), and other factors. Therefore, these factors should be taken into account when the characteristic elements of geographical origins were screened. At present, researchers are still studying the origin traceability technology in the feasibility study stage. There is no uniform standard method or procedure for establishing the discrimination model. Therefore, the systematic study on the origin tracing technology is significant for developing a standard method of origin tracing.

Quantity of samples directly affects statistical validity (Danezis and Georgiou 2022). For the setting of the quantity of samples, researchers can refer to Practical Guide to Chemometrics (Gemperline 2006). In the research on the origin traceability of agricultural products, relevant researchers have collected different sample quantities for the establishment of origin traceability models, and the quantity of modeling ranges from a dozen to hundreds, and the discriminative effects obtained are also different. For example, high‐resolution ICP‐MS was used to analyze the contents of mineral elements in 31 rice samples, and mineral elements in jasmine rice could be used to discriminate rice samples from different geographical origins correctly, with the overall correct discrimination rate of 90.3% (Pracha et al. 2014). ICP‐AES was used to determine the contents of mineral elements in 107 rice samples, and the overall correct discrimination rate of the corresponding origin tracing model established with those rice samples from different origins was 91.3% (Gonzalvez et al. 2009). The contents of mineral elements in 153 wine samples were determined by ICP‐MS, and the overall correct discrimination rate of the corresponding origin tracing model established with those wine samples from different regions reached 96.7% (Perez‐Trujill et al. 2003). The above studies indicated that there was no common requirement for the quantity of modeling samples. Therefore, it is necessary to experiment with the influence of sample quantity on discrimination results.

Based on the difference in the traceability scope of the samples used in the traceability model of agricultural product origin based on mineral element fingerprint analysis technology, the discriminative effect of establishing the origin discrimination model is also different. The contents of mineral elements in rice from different origins in China were determined with ICP‐OES and the accuracy of the origin tracing model established based on rice samples from different origins was 67.7% (Li et al. 2012). ICP‐AES and ICP‐HRMS are used to determine the content of mineral elements in rice samples from different origins in Japan. The results showed that the accuracy of the identification model based on different habitats was 96.3% (Akemi and Kumiko 2000). The contents of mineral elements in rice samples from different countries were determined with ICP‐MS and the accuracy of the corresponding tracing model was 100.0% (Pracha et al. 2013). The above studies showed that different traceability scopes of samples have different accuracy of geographical origin discrimination, so it is necessary to clarify the protection scope of specific products and the reference scope of other products.

In this study, with mung bean samples with geographical indications as the research object, the origin traceability models of mung beans were established under two conditions: different traceability scope and different quantities. The discrimination effects of the origin tracing models established with mung bean samples from Heilongjiang Province (Dorbod Mongol Autonomous, Tailai), Jilin Province (Baicheng), and Shandong Province (Sishui) were then compared. Sample traceability scope and sample quantity are important factors affecting the origin traceability model discrimination rate; this study, through the analysis of different traceability scopes and sample quantities of mung bean origin discrimination rates, illustrates the relationship between sample traceability scope, sample quantity, and origin discrimination rate. In summary, although existing research has confirmed that sample quantity and traceability scope have a key impact on the traceability model of mineral elements, the underlying laws are not yet clear. More importantly, previous research has confirmed that sample quantity has a decisive impact on the accuracy of agricultural product origin traceability models based on near‐infrared spectroscopy (Chen et al. 2024). However, the principles of different traceability technologies (such as near‐infrared spectroscopy and mineral element analysis) are very different, and the impact of the constructed models on the sample quantity and traceability scope may also be different. The near‐infrared spectrum reflects the vibration information of the functional groups of organic molecules, while the mineral element fingerprint reflects the minerals absorbed from the growth environment. Therefore, direct application of sample quantity standards derived from one technique to another lacks sufficient scientific basis.

This study aims to fill this knowledge gap. We take mineral element analysis technology as an example to explore its key issues: In element traceability technology, what are the similarities and differences between model performance changes as sample quantity increases and near‐infrared spectroscopy technology. Through this systematic comparative study, it has important theoretical and practical significance for formulating scientific and efficient sample collection and database construction plans for different traceability technologies.

## 2 Materials and Methods

### 2.1 Sample Collection

According to the principle of representative random sampling, mung bean samples were collected respectively in Baicheng City of Jilin Province (*n* = 30), Tailai County (*n* = 30), and Dorbod Mongol Autonomous County of Heilongjiang Province (*n* = 30), and Sishui County of Shandong Province (*n* = 30). Sampling sites were scaled according to the five parts of east, west, south, north, and middle. Sampling plots were set in each sampling site according to the planting scale, and 5 points were repeated on the diagonal of each sampling plot. Pods (1–3 kg) were collected at each sampling point. Detailed descriptions of sampling location, species, longitude, latitude (GPS, NEO‐6M‐0‐001, Shenzhen Pengrunfa Electronics Co. Ltd.), soil quality, and other data were provided in Table 1.

**TABLE 1:** The sample numbers, location, and soil property of mung beans geographical origins.

Sampling location	Year	Sampling number	Species	Soil property	Average annual temperature	Longitude (E)	Latitude (N)
Baicheng City, Jilin Province	2020	30	Brother green, small green, longbo 9, hairy mung beans	Light black calcium soil, meadow soil	6.0°C	122°38′–123°22′	45°13′–45°18′
	2021	30	Little rotgreen, hairy mung beans				
Tailai County, Heilongjiang Province	2020	30	Bright mung beans, yellow mung beans, hairy mung beans, rotgreen	Sandy loam soil, meadow soil	4.5°C	123°42′–124°20′	46°39′–46°59′
	2021	30	Mao mung beans, bright mung beans				
Dorbod Mongol Autonomous County, Heilongjiang Province	2020	30	Brother green, small green, longbo 9, hairy mung beans	Meadow soil, sand soil	6.0°C	124°44′–124°46′	47°26′–47°36′
	2021	30	Little rotgreen, hairy mung beans				
Sishui County, Shandong Province	2020	30	Bright mung beans, yellow mung beans, hairy mung beans, rotgreen	Brown loam soil, cinnamon soil	13.4°C	117°09′–117°15′	35°23′–35°39′
	2021	30	Mao mung beans, bright mung beans				

### 2.2 Sample Preparation

The collected pods were numbered according to sampling sites and dried in a dust‐free, clean, and sunless place. After impurities such as shell, dust, and small stone particles were removed, complete mung bean seeds were collected, rinsed with deionized water to remove dust, dried in an oven (DGG‐923A, Shanghai Jinghong Experimental Equipment Co. Ltd.) at 38°C until the moisture content was below 13%. The dried mung beans were pulverized with a whirlwind mill (CT193CyclotecTM, Guangzhou Easy Test Instrument Co. Ltd.). Mung bean powder was obtained through a 60–mesh nylon sieve and stored in the polyethylene bags in a desiccator until analysis. All samples were treated in the same manner.

### 2.3 Sample Digestion and Determination Method of Element Content

Firstly, 0.500 g mung bean powder sample was accurately weighed and placed in a teflon tube. Then, 10 mL concentrated nitric acid (70%, BV3 grade, J.T. Baker Ltd., USA) was added in the tube to digest the sample in a microwave digestion instrument (MARS6classical, American GEM). Microwave digestion program was set as follows: heated from room temperature to 120°C within 5 min, maintained for 5 min, heated to 150°C within 5 min, maintained for 10 min, heated to 190°C within 5 min, maintained for 15 min, and then cooled for 20 min. After the digestion procedure was completed, the microwave tube was taken out. After the plug was opened in the fume hood, the microwave digestion tube was placed in an accurately temperature‐controlled electrothermal digester (EHD‐24, Beijing East Aviation Science Instrument Co. Ltd.) for 60 min acid removal at 120°C. After cooling to room temperature, the digestion solution was diluted to a volume of 50 mL with ultra‐pure water (SMARt‐N, > 18.2 MΩ•cm Shanghai Conray Analysis Instrument Co. Ltd.) for subsequent detection. Before dilution, residual weight in the tube was about 10–11 g. Different elements were diluted according to the linear scale of element standard curve. The blank sample and standard material were digested in the same manner (He et al. 2020).

The operating parameters of ICP‐MS (7700, Agilent, USA) were set as follows: RF power of 1550 W, atomization chamber temperature of 2°C, peristaltic pump speed of 0.10 RPS, the carrier gas flow rate of 0.9 L/min, the plasma gas flow rate of 15.0 L/min, Helium flow rate of 4.3 mL/min, sampling depth of 0.80 cm, and full quantitative mode.

The contents of 52 elements (Na, Mg, Al, K, Ca, Sc, V, Cr, Mn, Fe Co, Ni, Cu, Zn, As, Se, Rb, Sr., Y, Mo, Ru, Rh, Pd, Ag, Cd, Sn, Sb, Te, Cs, Ba, La, Ce, Pr, Nd, Sm, Eu, Gd Tb, Dy, Ho, Er, Tm, Yb, Lu, Hf, Ir, Pt, Au, Tl, Pb, Th, and U) were determined with ICP‐MS. In the determination process, the element recovery of the standard was required to be greater than 90%.

In the determination process, each sample was determined for 3 times and the three data were averaged. Ge, In, and Bi elements were selected as internal standard elements (Agilent) to reflect the stability of the instrument. If the relative standard deviations (RSD) of internal standard elements were greater than 5%, the sample would be re‐determined.

### 2.4 Data Processing

SPSS Statistics 27.0 software was used to perform the variation analysis (Duncan multiple comparison test) and discriminant analysis (Fisher's test) with the data obtained from ICP‐MS.

## 3 Results

### 3.1 Analysis of the Difference in the Contents of Mineral Elements in Mung Beans From Different Geographical Origins and Years

The influences of different geographical origins on the contents of mineral elements in mung beans were studied through the variance analysis of mung bean samples from Dorbod Mongol Autonomous, Baicheng, Tailai, and Sishui. The analysis results showed that the contents of Na, Mg, P, K, Ca, B, Fe, Al, Mn, Cu, Zn, As, Mo, Ag, Ba, Rb, Sr., V Co, Se, and Pd were significantly different among the samples from different geographical origins (*p* < 0.05) (Table 2). These differences might be due to the unique geographical environment such as climate, hydrology, and soil type. The contents of mineral elements in mung beans were affected by the special geographical environment, so the contents of mineral elements in mung beans from different geographical origins were significantly different (Buggle et al. 2008). The effects of different years (2020, 2021) on the content of mineral elements in mung bean samples from different origins were compared through variance analysis (Table 3). The results showed that there were significant differences in the contents of B, Na, Mg, P, K, Ca, V, Mn Co, As, Rb, Cd, Cs, Ba, Lu, Pt, and Pb among samples from different years (*p* < 0.05). Further analysis found that the trends in origin differences of elements such as Na, P, K, As, Ba, and Cd in different years (2020, 2021) are consistent. For example, the As content showed that Tailai > Sishui > Dorbod Mongol Autonomous and Baicheng. This shows that the differences in the content of these elements are mainly affected by the geographical origins (soil type, climate conditions), but are less affected by changes in years, and can be used as characteristic indicators to build a stable origin traceability model. Elements such as B, Rb, Cs, and V have changed between different years (2020, 2021). For example, the Rb element appears in the data of different years (2020, 2021) as: Tailai and Baicheng > Dorbod Mongol Autonomous and Sishui. This shows that although the year will affect its elemental content, it can still effectively reflect the characteristics of the geographical origins. Elements such as Pb, Lu, and Pt have undergone significant changes in the 2 years with different geographical origins. For example, the content of Pb in Baicheng samples increased abnormally from a medium level (102.9 μg/kg) in 2020 to an extremely high value (867.6 μg/kg) in 2021. This is most likely due to accidental contamination of individual samples or anomalies in specific years. Therefore, by systematically comparing the mineral element contents in different years, characteristic mineral elements that are less affected by year and have stability are selected, which provides a scientific basis for establishing a stable mineral element traceability database in different years.

**TABLE 2:** Mineral element content in mung bean from different origins.

Mineral element	Tailai	Dorbod Mongol Autonomous	Baicheng	Sishui
Na (g/kg)	0.69 ± 0.11^a^	0.42 ± 0.08^b^	0.47 ± 0.12^b^	0.67 ± 0.01^a^
Mg (g/kg)	1.15 ± 0.06^a^	1.21 ± 0.05^a^	0.98 ± 0.07^c^	1.13 ± 0.01^b^
P (g/kg)	6.09 ± 0.35^ab^	5.83 ± 0.26^b^	5.03 ± 0.64^c^	6.31 ± 0.03^a^
K (g/kg)	8.69 ± 0.45^b^	8.91 ± 0.44^b^	7.55 ± 0.59^c^	9.59 ± 0.10^a^
Ca (g/kg)	0.61 ± 0.09^ab^	0.62 ± 0.08^a^	0.63 ± 0.09^a^	0.59 ± 0.03^b^
B (mg/kg)	13.18 ± 1.53^b^	14.18 ± 1.63^b^	11.88 ± 1.76^c^	16.08 ± 1.06^a^
Fe (mg/kg)	27.51 ± 2.32^ab^	31.09 ± 1.56^a^	27.86 ± 2.76^ab^	11.46 ± 1.04^b^
Al (mg/kg)	3.95 ± 0.92^a^	2.16 ± 0.80^ab^	1.88 ± 1.02^ab^	1.39 ± 0.24^b^
Mn (mg/kg)	12.33 ± 2.11^a^	11.49 ± 1.40^a^	7.52 ± 0.69^b^	7.30 ± 0.11^b^
Cu (mg/kg)	4.53 ± 1.29^c^	4.39 ± 1.32^c^	6.12 ± 1.45^b^	7.88 ± 2.15^a^
Zn (mg/kg)	19.62 ± 3.83^c^	21.03 ± 4.12^bc^	22.55 ± 3.70^ab^	24.43 ± 3.62^a^
As (mg/kg)	0.15 ± 0.03^a^	0.10 ± 0.01^b^	0.13 ± 0.02^b^	0.13 ± 0.00^a^
Mo (mg/kg)	1.32 ± 0.71^b^	2.01 ± 0.38^ab^	2.35 ± 0.45^a^	2.39 ± 0.12^a^
Ag (mg/kg)	0.24 ± 0.03^a^	0.27 ± 0.14^ab^	0.15 ± 0.02^b^	0.24 ± 0.15^ab^
Ba (mg/kg)	1.41 ± 0.42^ab^	1.15 ± 0.55^b^	0.80 ± 0.16^c^	1.68 ± 0.03^a^
Rb (mg/kg)	5.95 ± 1.29^a^	3.49 ± 1.18^b^	5.78 ± 1.41^a^	2.58 ± 0.02^b^
Sr (mg/kg)	5.44 ± 1.08^a^	3.87 ± 1.19^ab^	5.50 ± 2.59^a^	3.12 ± 0.08^b^
V (μg/kg)	41.54 ± 5.39^a^	16.23 ± 6.64^b^	15.80 ± 0.77^b^	14.17 ± 3.91^b^
Co (μg/kg)	42.31 ± 20.42^a^	38.14 ± 12.75^a^	25.12 ± 8.41^b^	33.86 ± 3.41^ab^
Se (μg/kg)	26.32 ± 1.92^a^	19.83 ± 3.86^ab^	15.23 ± 5.73^b^	22.37 ± 8.90^ab^
Pd (μg/kg)	0.65 ± 0.43^b^	1.21 ± 0.65^b^	3.38 ± 2.29^a^	0.23 ± 0.25^b^

*Note:* Lowercase letters indicate significant differences (*p* < 0.05).

**TABLE 3:** Mineral element content in mung bean from 2020 and 2021.

Mineral element	2020				2021			
	Tailai (*n* = 30)	Dorbod Mongol Autonomous (*n* = 30)	Baicheng (*n* = 30)	Sishui (*n* = 30)	Tailai (*n* = 30)	Dorbod Mongol Autonomous (*n* = 30)	Baicheng (*n* = 30)	Sishui (*n* = 30)
B/(mg/kg)	13.11 ± 1.38^b^	13.90 ± 1.63^b^	11.72 ± 1.98^c^	15.8 ± 1.1^a^	13.35 ± 1.98^b^	15.75 ± 1.16^a^	11.85 ± 1.45^c^	16.07 ± 0.91^a^
Na/(mg/kg)	685.3 ± 114.8^a^	404.2 ± 18.0^b^	394.6 ± 20.8^b^	658.5 ± 11.2^a^	662.4 ± 14.7^a^	647.0 ± 28.1^c^	656.1 ± 14.4^b^	662.2 ± 9.7^a^
Mg/(mg/kg)	1191.1 ± 89.3^a^	1214.2 ± 59.8^a^	1007.7 ± 51.8^b^	1108.3 ± 12.5^ab^	1203.0 ± 42.1^a^	1214.9 ± 57.7^a^	976.7 ± 99.8^b^	1115.1 ± 8.4^c^
P/(mg/kg)	5138.4 ± 382.5^ab^	4772.4 ± 279.0^b^	3897.4 ± 686.3^c^	5285.4 ± 40.2^a^	5203.1 ± 278.5^a^	4990.3 ± 212.3^b^	4186.6 ± 533.9^c^	5309.0 ± 31.5^a^
K/(mg/kg)	8674.3 ± 505.7^b^	8846.2 ± 438.7^b^	7531.7 ± 512.8^c^	9540.2 ± 125.6^a^	9026.8 ± 223.6^b^	9123.7 ± 311.7^b^	7665.4 ± 628.7^c^	9592.5 ± 107.0^a^
Ca/(mg/kg)	587.5 ± 103.9^c^	641.1 ± 83.2^a^	627.0 ± 77.8^b^	571.2 ± 35.6^c^	529.7 ± 23.2^b^	645.1 ± 112.3^a^	649.2 ± 108.7^a^	576.4 ± 30.1^b^
V/(μg/kg)	47.7 ± 56.9^a^	16.4 ± 9.2^b^	16.2 ± 8.5^b^	15.2 ± 5.5^b^	23.1 ± 4.7^a^	15.6 ± 3.4^b^	18.3 ± 9.8^ab^	16.17 ± 4.99^b^
Mn/(μg/kg)	11502.6 ± 2327.4^a^	10451.2 ± 1714.1^a^	8502.8 ± 982.5^b^	8250.3 ± 210.5^b^	11102.9 ± 2004.0^a^	9308.9 ± 756.1^b^	8005.5 ± 1018.6^c^	8297.0 ± 189.0^c^
Co/(μg/kg)	40.5 ± 22.3^a^	37.9 ± 15.7^a^	26.6 ± 11.5^b^	30.8 ± 15.2^ab^	29.7 ± 8.6^a^	30.7 ± 7.8^a^	17.2 ± 4.9^b^	32.96 ± 13.98^a^
As/(μg/kg)	182.6 ± 40.9^a^	113.9 ± 13.6^b^	100.6 ± 7.9^b^	161.8 ± 3.5^c^	162.9 ± 4.0^a^	158.3 ± 10.1^b^	159.0 ± 13.4^b^	161.45 ± 2.78^a^
Rb/(μg/kg)	5583.6 ± 2074.3^a^	3225.7 ± 1247.4^b^	5222.9 ± 1515.9^a^	2490.5 ± 70.3^b^	4839.4 ± 1758.6^ab^	4170.7 ± 758.5^b^	5809.3 ± 2835.2^a^	2524.3 ± 59.6^c^
Cd/(μg/kg)	5.47 ± 10.27^a^	3.43 ± 2.46^b^	2.0 ± 1.2^c^	1.65 ± 0.40^c^	2.79 ± 1.37^a^	1.92 ± 1.18^b^	1.09 ± 0.65^c^	1.73 ± 0.33^b^
Cs/(μg/kg)	25.5 ± 17.5^a^	6.8 ± 5.4^b^	14.5 ± 7.2^c^	2.2 ± 0.9^d^	17.2 ± 13.3^a^	11.4 ± 2.4^b^	17.2 ± 16.8^a^	2.45 ± 1.04^c^
Ba/(μg/kg)	1361.6 ± 588.9^ab^	1266.3 ± 672.1^ab^	857.7 ± 298.8^b^	1620.8 ± 90.2^a^	1409.4 ± 530.4^b^	1088.4 ± 443.1^c^	908.6 ± 421.6^d^	1646.6 ± 80.4^a^
Lu/(μg/kg)	265.3 ± 147.7^b^	494.6 ± 68.0^a^	419.5 ± 119.8^a^	158.2 ± 110.5^b^	272.9 ± 166.6^a^	258.5 ± 65.7^a^	127.7 ± 94.7^b^	165.5 ± 120.1^ab^
Pt/(μg/kg)	1.23 ± 0.39^b^	1.74 ± 0.72^a^	1.6 ± 0.9^a^	1.05 ± 0.30^c^	0.75 ± 0.19^c^	1.16 ± 0.25^b^	1.33 ± 0.49^a^	1.10 ± 0.26^b^
Pb/(μg/kg)	77.1 ± 88.2^c^	63.0 ± 32.3^c^	102.9 ± 63.0^b^	135.5 ± 120.8^a^	178.4 ± 84.2^b^	98.4 ± 50.3^c^	867.6 ± 1490.9^a^	149.9 ± 145.4^b^

*Note:* Lowercase letters indicate significant differences (*p* < 0.05).

### 3.2 Influences of Sample Scale on the Origin Tracing Model of Mung Beans

#### 3.2.1 Influences of the Scales From Different Geographical Origins on Discrimination Rate

**FIGURE 1:**
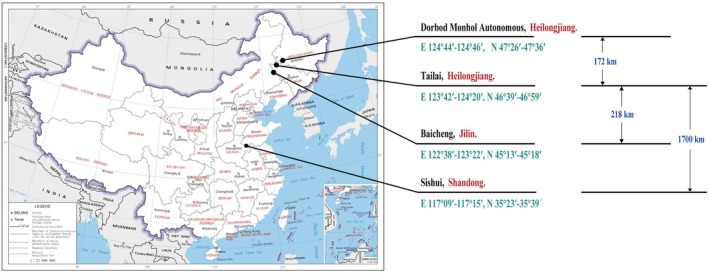
Actual geographical scale map of mung bean samples from different geographical origins.

In order to further analyze the influences of the scales from different geographical origins on discrimination results, based on the screened mineral elements related to the origin as the tracing index, mung beans from Tailai were taken as the starting point and three tracing scales from Tailai to different sites were set as follows: Talai–Dorbod Mongol Autonomous (short scale), Tailai–Baicheng (medium scale), Tailai–Sishui (long scale). Samples from different sites with short, medium, and long tracing scales were respectively labeled as Groups A, B, and C. The origin discriminant analysis was carried out on the three groups of mung beans from near, medium and far, and three groups of classification recognition functions and classification results corresponding to the origin were obtained. The results showed the overall accuracy and the cross–validation rate of Discrimination Model A established based on the samples from Tailai and Dorbod Mongol Autonomous were respectively 99.33% and 96.67%. The overall accuracy and the cross–validation rate of Discrimination Model B established based on the samples from Tailai and Baicheng were respectively 99.67% and 99.33%. The overall accuracy and the cross–validation rate of Discrimination Model C established based on the samples from Tailai and Sishui were respectively 100.0% and 100.0% (Table 4). Overall discrimination accuracy refers to the percentage of the sample quantity in all localities and the actual sample quantity in all localities. Cross–validation is a common method in machine learning to build models and verify model parameters. It means to divide the obtained data and combine for the different training sets and test sets. The training set is used to train the model, and the test set is used to evaluate the model predictions. The accuracies of Discrimination Models (A, B, and C) with different tracing scales were increased according to the following order: Discrimination Model A (Tailai–Dorbod Mongol Autonomous) < Discrimination Model B (Tailai–Baicheng) = Discrimination Model C (Tailai–Sishui), namely, 99.33% < 99.67% = 100.0%. The cross–validation rate of Discrimination Models (A, B, and C) with different tracing scales were increased according to the following order: Discrimination Model A (Tailai–Dorbod Mongol Autonomous) < Discrimination Model B (Tailai–Baicheng) < Discrimination Model C (Tailai–Sishui), namely, 96.67% < 99.33% < 100.0%. The tracing scales of the samples in three groups were increased according to the following order: Group A (Tailai–Dorbod Mongol Autonomous) < Group B (Tailai–Baicheng) < Group C (Tailai–Sishui). It showed that the discrimination rate is proportional to the scale of the sample origin, and the geographical scale of the sample source has an impact on the discrimination rate of the model. The larger the scale, the higher the correct discrimination rate. Actual scale map for samples of different groups (Figure 1).

**TABLE 4:** Classification results of mung beans from different origin in three groups A, B, and C^a^, ^b^, ^c^, ^d^, ^e^, ^f^, ^g^.

Tracing scale	Group		Sampling location	Prediction group member information				Total
				Dorbod Mongol Autonomous	Tailai	Baicheng	Sishui	
A	Original	Count	Dorbod Mongol Autonomous	149	1	—	—	150
			Tailai	1	149	—	—	150
		Determination rate/%	Dorbod Mongol Autonomous	99.33	0.67	—	—	100.0
			Tailai	0.67	99.33	—	—	100.0
	Cross validation^b^	Count	Dorbod Mongol Autonomous	145	5	—	—	150
			Tailai	5	145	—	—	150
		Determination rate/%	Dorbod Mongol Autonomous	96.67	3.33	—	—	100.0
			Tailai	3.33	96.67	—	—	100.0
B	Original	Count	Tailai	—	150	0	—	150
			Baicheng	—	1	149	—	150
		Determination rate/%	Tailai	—	100.0	100.0	—	100.0
			Baicheng	—	0.67	99.33	—	100.0
	Cross validation^b^	Count	Tailai	—	149	1	—	150
			Baicheng	—	1	149	—	150
		Determination rate/%	Tailai	—	99.33	0.67	—	100.0
			Baicheng	—	0.67	99.33	—	100.0
C	Original	Count	Tailai	—	150	—	0	150
			Sishui	—	0	—	150	150
		Determination rate/%	Tailai	—	100.0	—	0.0	100.0
			Sishui	—	0.0	—	100.0	100.0
	Cross validation^b^	Count	Tailai	—	150	—	0	150
			Sishui	—	0	—	150	150
		Determination rate/%	Tailai	—	100.0	—	0.0	100.0
			Sishui	—	0.0	—	100.0	100.0

^a^ 99.33% of the samples in the initial grouping case have been correctly classified.

^b^ Only cross–validate the cases in the analysis. In cross–validation, each case is classified according to a function derived from all other cases except that case.

^c^ 96.67% of the samples in the cross–validation grouping cases have been correctly classified.

^d^ 99.67% of the samples in the initial grouping case have been correctly classified.

^e^ 99.33% of the samples in the cross–validation grouping cases have been correctly classified.

^f^ 100.0% of the samples in the initial grouping case have been correctly classified.

^g^ 100.0% of the samples in the cross–validation grouping cases have been correctly classified.

#### 3.2.2 Discriminant Analysis of the Origin of Mung Bean With Different Traceability Scope

In order to further study the influences of sample scale on discrimination results, 21 mineral elements related to geographical origin were introduced. Discriminant analysis was carried out respectively with the samples from two traceability scopes, including one small scale and one large scale. The small scale covered Tailai, Dorbod Mongol Autonomous, and Baicheng. The large scale covered the Northeast region and Shandong Province.

**FIGURE 2:**
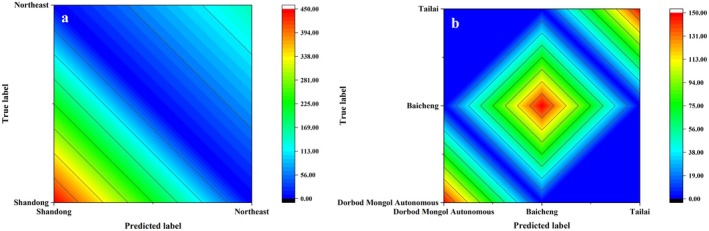
Contour map of mung bean origin discrimination at different traceability scope. (a) Contour map of mung bean provenance discrimination on a larger scale (Northeast–Shandong). (b) Contour map of mung bean provenance discrimination on a smaller scale (Dorbod Mongol Autonomous–Baicheng–Tailai).

Fisher's criterion in SPSS discriminant analysis was adopted. The 21 mineral elements related to the geographical origin were introduced by inputting independent variables together. The discriminant analysis of mung beans from large and small scales was carried out in the cross–validation mode. The classification discrimination functions and classification results of large and small scales were obtained. The overall accuracy and cross–validation rate of the model established with the samples from the small scale were respectively 99.55% and 98.0%. The overall accuracy and cross–validation rate of the model established with the samples from the large scale were respectively 100.0% and 99.89% (Table 5). Contour map of mung bean origin discrimination at different traceability scope (Figure 2). The above results indicated that the overall accuracy of the model established with the samples from the large scale was higher than that of the model established with the samples from the small scale. The larger scale between the modeling samples can improve the accuracy of the model, which may be related to the influence of various factors such as climate, light, and precipitation in different regions. Learned from information, the small scale (northeast region) is characterized by temperate continental monsoon climate, annual average sunshine duration of about 2882.2 h, and average annual precipitation of about 397.5 mm. The large scale (Shandong Province) is characterized by a warm temperate monsoon climate, annual sunshine percentage of 52%, and average annual precipitation of about 760 mm. It can be seen that the composition and content of mineral elements carried by the plants are also quite different due to the large differences in the growth environment between the samples from the large–scale geographical origins. In addition, the geographical environment within the small scale was similar, so the discrimination effect was worse than that of the samples from the large scale. Therefore, it is necessary to add new origin fingerprint information in origin tracing of agricultural products in order to improve the discrimination rate of samples from a small scale.

**TABLE 5:** Classification results of mung bean from large and small scales ^a^, ^b^, ^c^, ^d^, ^e^.

Group		Sampling location	Prediction group member information					Total
			Northeast mung bean	Shandong mung bean	Dorbod Mongol Autonomous	Baicheng	Tailai	
Original	Count	Northeast mung bean	450	0	—	—	—	450
		Shandong mung bean	0	150	—	—	—	150
	Determination rate/%	Northeast mung bean	100.0	0	—	—	—	100.0
		Shandong mung bean	0	100.0	—	—	—	100.0
Cross validation^b^	Count	Northeast mung bean	449	1	—	—	—	450
		Shandong mung bean	0	150	—	—	—	150
	Determination rate/%	Northeast mung bean	99.78	0.22	—	—	—	100.0
		Shandong mung bean	0.0	100.0	—	—	—	100.0
Original	Count	Dorbod Mongol Autonomous	—	—	149	0	1	150
		Baicheng	—	—	0	150	0	150
		Tailai	—	—	1	0	149	150
	Determination rate/%	Dorbod Mongol Autonomous	—	—	99.33	0.0	0.67	100.0
		Baicheng	—	—	0.0	100.0	0.0	100.0
		Tailai	—	—	0.67	0.0	99.33	100.0
Cross validation^b^	Count	Dorbod Mongol Autonomous	—	—	145	1	4	150
		Baicheng	—	—	0	149	1	150
		Tailai	—	—	3	0	147	150
	Determination rate/%	Dorbod Mongol Autonomous	—	—	96.67	0.67	2.66	100.0
		Baicheng	—	—	0.0	99.33	0.67	100.0
		Tailai	—	—	2.0	0.0	98.00	100.0

^a^ 100.0% of the samples in the initial grouping case have been correctly classified.

^b^ Only cross–validate the cases in the analysis. In cross–validation, each case is classified according to a function derived from all other cases except that case.

^c^ 99.89% of the samples in the cross–validation grouping cases have been correctly classified.

^d^ 99.55% of the samples in the initial grouping case have been correctly classified.

^e^ 98.0% of the samples in the cross–validation grouping cases have been correctly classified.

### 3.3 Influences of Sample Quantity on the Discrimination Results of Origin Tracing Models of Mung Beans

The discriminant analysis was performed respectively with 200 (Group A), 400 (Group B), and 600 (Group C) samples from different geographical origins. Firstly, 50, 100, and 150 samples were selected from four geographical origins of three groups (A, B, and C). Based on the differential elements among different geographical origins and SPSS discriminant analysis of Fisher's criterion, the influences of the quantity of modeling samples on the discrimination results were analyzed. The classification functions and discrimination results of Groups A, B, and C were obtained. The results showed that the overall correct discrimination rate of Group A (*n* = 200) was 99.0%, and the cross–validation rate was 97.5%. The overall accuracy and cross–validation rate of Group B (*n* = 400) were respectively 99.25% and 97.75%. The overall accuracy and cross–validation rate of Group C (*n* = 600) were respectively 99.33% and 98.65%. The overall accuracy and cross–validation rate of 600 samples in Group C were the highest. The order of the overall correct discrimination rate and the established model cross–validation discrimination rate for discriminant analysis with different sample quantities is C (*n* = 600) > B (*n* = 400) > A (*n* = 200) (Table 6).

**TABLE 6:** Classification results of mung bean origin of different sample quantities^a^, ^b^, ^c^, ^d^, ^e^, ^f^, ^g^.

Sample quantity	Group		Sampling location	Prediction group member information				Total
				Dorbod Mongol Autonomous	Baicheng	Tailai	Sishui	
A	Original	Count	Dorbod Mongol Autonomous	49	0	1	0	50
			Baicheng	0	50	0	0	50
			Tailai	1	0	49	0	50
			Sishui	0	0	0	50	50
		Determination rate/%	Dorbod Mongol Autonomous	98.0	0.0	2.0	0.0	100.0
			Baicheng	0.0	100.0	0.0	0.0	100.0
			Tailai	2.0	0.0	89.0	0.0	100.0
			Sishui	0.0	0.0	0.0	100.0	100.0
	Cross validation^b^	Count	Dorbod Mongol Autonomous	47	0	3	0	50
			Baicheng	0	49	1	0	50
			Tailai	1	0	49	0	50
			Sishui	0	0	0	50	50
		Determination rate/%	Dorbod Mongol Autonomous	94.0	0.0	6.0	0.0	100.0
			Baicheng	0.0	98.0	2.0	0.0	100.0
			Tailai	2.0	0.0	98.0	0.0	100.0
			Sishui	0.0	0.0	0.0	100.0	100.0
B	Original	Count	Dorbod Mongol Autonomous	98	0	2	0	100
			Baicheng	0	100	0	0	100
			Tailai	1	0	99	0	100
			Sishui	0	0	0	100	100
		Determination rate/%	Dorbod Mongol Autonomous	98.0	0.0	2.0	0.0	100.0
			Baicheng	0.0	100.0	0.0	0.0	100.0
			Tailai	1.0	0.0	99.0	0.0	100.0
			Sishui	0.0	0.0	0.0	100.0	100.0
	Cross validation^b^	Count	Dorbod Mongol Autonomous	95	0	4	1	100
			Baicheng	0	100	0	0	100
			Tailai	3	0	96	1	100
			Sishui	0	0	0	100	100
		Determination rate/%	Dorbod Mongol Autonomous	95.0	0.0	4.0	1.0	100.0
			Baicheng	0.0	100.0	0.0	0.0	100.0
			Tailai	3.0	0.0	96.0	1.0	100.0
			Sishui	0.0	0.0	0.0	100.0	100.0
C	Original	Count	Dorbod Mongol Autonomous	147	0	3	0	150
			Baicheng	0	150	0	0	150
			Tailai	1	0	149	0	150
			Sishui	0	0	0	150	150
		Determination rate/%	Dorbod Mongol Autonomous	98.0	0.0	2.0	0.0	100.0
			Baicheng	0.0	100.0	0.0	0.0	100.0
			Tailai	0.7	0.0	99.3	0.0	100.0
			Sishui	0.0	0.0	0.0	100.0	100.0
	Cross validation^b^	Count	Dorbod Mongol Autonomous	145	1	4	0	150
			Baicheng	0	149	1	0	150
			Tailai	1	0	148	1	150
			Sishui	0	0	0	150	150
		Determination rate/%	Dorbod Mongol Autonomous	96.7	0.7	2.6	0.0	100.0
			Baicheng	0.0	99.3	0.7	0.0	100.0
			Tailai	0.7	0.0	98.6	0.7	100.0
			Sishui	0.0	0.0	0.0	100.0	100.0

^a^ 99.0% of the samples in the initial grouping case have been correctly classified.

^b^ Only cross‐validate the cases in the analysis. In cross‐validation, each case is classified according to a function derived from all other cases except that case.

^c^ 97.5% of the samples in the cross‐validation grouping cases have been correctly classified.

^d^ 99.25% of the samples in the initial grouping case have been correctly classified.

^e^ 97.75% of the samples in the cross‐validation grouping cases have been correctly classified.

^f^ 99.33% of the samples in the initial grouping case have been correctly classified.

^g^ 98.65% of the samples in the cross‐validation grouping cases have been correctly classified.

**FIGURE 3:**
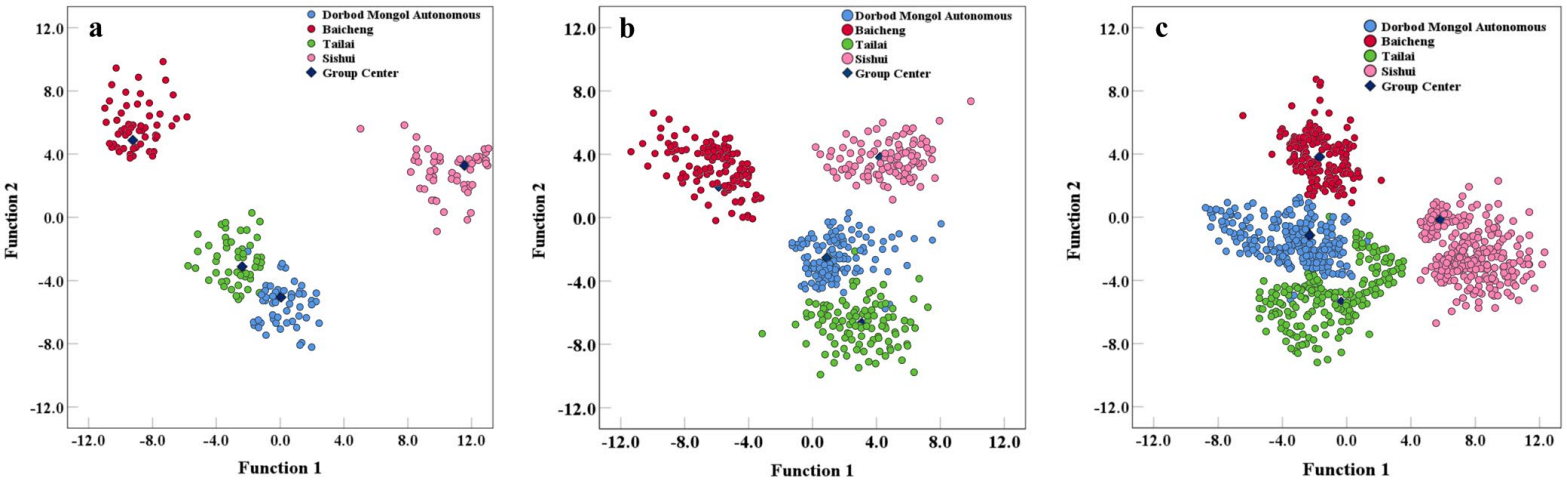
Scoplot of mung bean discrimination function in different origin. (a) Discriminant function score plot for the number of 40 samples. (b) Discriminant function score plot for the number of 80 samples. (c) Discriminant function score plot for the number of 120 samples.

With the scores of discrimination functions, the distribution map of mung bean samples from different geographical origins in Group A (*n* = 200), Group B (*n* = 400), and Group C (*n* = 600) was plotted (Figure 3). It can be seen from the figure that the discriminant functions of different sample quantities can distinguish samples from different origins, and the discrimination effect is C (*n* = 600) > B (*n* = 400) > A (*n* = 200). The accuracy of discriminant analysis was improved gradually when sample quantity in discriminant analysis increased within a certain scale, indicating that sample quantity had an impact on the origin tracing model. A large sample quantity could represent the geographical scope more accurately, thus improving the stability and accuracy of the discrimination model.

## 4 Discussion

Since the mineral elements in agricultural products cannot be synthesized and ingested from the surrounding environment, their content is closely related to the water, soil, climate and other planting areas, and different regions have their own element composition characteristics. Thus forming a fingerprint of the mineral elements of agricultural products with different sources of origins (Karami et al. 2009). In this paper, we studied the mineral element composition and content differences derived from mung bean Tailai, Dorbod Mongol Autonomous County in Heilongjiang Province, Baicheng City in Jilin Province, and Sishui County in Shandong Province. The mineral elements of mung bean in different origins have their own characteristics, mainly affected by the natural environment such as soil and climate. Baicheng City, Jilin Province belongs to temperate continental climate; Tailai and Dorbod Mongol Autonomous County, Heilongjiang Province belong to the cold temperate continental monsoon climate, Sishui County, Shandong Province belongs to warm‐temperate monsoon climate. The differences in climate showed different annual temperatures and precipitation, which influenced the uptake of elements. The average Baicheng City in Jilin Province annual temperature in northeast China is 6.0°C (2020–2021), and the soil types are mostly light black calcium and meadow soil; the average annual temperature in Dorbod Mongol Autonomous County in Heilongjiang Province years is 6.0°C (2020–2021), and the soil type is mostly meadow and sand soil; the average annual temperature in Tailai County in Heilongjiang Province years is 4.5°C (2020–2021), and the soil types are mostly meadow and sandy loam soil; the annual average temperature of Shandong Province (Sishui) is 13.4°C (2020–2021), and the soil types are brown loam and cinnamon soil. As the soil types and the annual average temperature of the three geographical origins in northeast China are similar, which is the main reason for the small difference in mineral elements in the small geographical origins in northeast China. It increases the difficulty of tracing the origin of mung bean in a small scale, while the annual average temperature between the three geographical origins in Shandong province and that in northeast China is much different, so the mineral element content of mung bean in Sishui, Shandong province is much more different.

In order to deeply analyze the internal mechanism that affects the formation of elemental fingerprints, this study used variance analysis to screen out the characteristic mineral elements (such as Sr., Ba, Zn, V Co, etc.) that distinguish mung bean samples from different origins. The differences in the content of these elements are closely related to the climatic conditions, soil types and agricultural practices in different geographical origins (Yuan et al. 2012). Among them, the contents of elements such as Sr. and Ba are mainly affected by the soil parent material, which can effectively reflect the spatial heterogeneity of the regional geological background (Li et al. 2014); Zn is an essential nutrient element for plants, and its accumulation process is jointly regulated by the soil background content and artificial fertilization activities (Zhang, Nie, et al. 2022); the distribution of trace elements such as V and Co in soil is often associated with specific rock types and weathering processes, and their bioavailability is also significantly affected by soil pH and redox conditions (Aubin et al. 2025). To sum up, the discriminant effect of the model not only depends on the screening of characteristic elements, but is also affected by the differences in environmental conditions and agricultural practices covered by the sample (Supriya et al. 2021). For example, climate conditions, soil type (soil pH, organic matter content), etc. indirectly affect the mineral element fingerprint in the sample by regulating the migration and enrichment of elements in the soil–plant system (Zhang et al. 2020). In addition, differences in fertilizer application (such as Cd, As and other elements associated with phosphate fertilizers) and irrigation water sources in different origins may also introduce element signals with origin characteristics (Hill et al. 2024). This also explains to a certain extent why the model shows better discrimination results in large‐scale origin discrimination studies where climate and soil types vary significantly. Using barley elements from different Danish Sand and Loamy sand, the results showed that different soils had great effects on Fe, Mn, Cu, Zn, and Ni elements in barley (Husted et al. 2004). Consistent with the significant differences in the element content of Fe, Mn, Cu, and Zn in this study, it may be related to the different soil types in different origin. In addition, the contents of mineral elements in mung bean samples were also affected by factors such as variety and year. For example, Qian et al. (2023).

The selection of sample tracing scales will affect the discrimination effect of origin traceability, and the climate and geological types of the large scope of origin traceability will vary greatly, resulting in obvious differences in the indexes of selected mineral elements. He et al. (2020) used mineral element analysis techniques and analyzed the influence of large scale (Peru, China) and small scale (Yunnan, Xinjiang, Tibet) on origin identification results. The overall identification accuracy of the large scale (Peru and China) is 96.2%. For the overall identification accuracy of maca from a small scale (Yunnan, Xinjiang, Tibet) is 80.2% (He et al. 2020). Kaoru et al. (2012) used high‐resolution inductively coupled plasma mass spectrometry to determine the mineral element content in rice from a large area (Japan, Thailand, the United States, China). The results showed that the discrimination accuracy rate of the origin traceability model based on different origin is 97.0% (Kaoru et al. 2012). Shi et al. used mineral element fingerprint technology to determine the mineral element content in rice from small areas (Songjiang and Jinshan and Chongming); the results showed that the discrimination accuracy of the origin traceability model based on different origin is 92.1% (Shi et al. 2020). The above studies' conclusions are consistent with the conclusions of this study. The traceability scope of samples from different origins is related to the accuracy of origin traceability. The larger the origin traceability scope, the higher the discrimination accuracy. Due to the large differences in the origin, climate, soil, water, and other factors among a large scale of samples, the screening mineral element indicators had obvious differences, and the traceability effect was better. However, further experiments were still needed to confirm this point to give more accurate suggestions for establishing the origin traceability method of mineral elements in agricultural products. Similarly, we found that when the researchers established the mineral element origin traceability method, the quantity of samples collected was different, and the quantity of samples directly affected the discriminative accuracy of the origin traceability model. It has been shown that studies base their conclusions on a small number of samples, usually less than 100 (Danezis and Georgiou 2022). When a more accurate discriminative model is established, more sample quantities are needed (Zhang, Sun, et al. 2022). This study confirmed this by designing the influence study of different sample quantities on establishing the accuracy of origin discrimination. The more the quantity of samples, the closer the representative of its origin.

There were also some other methods for mung bean geographical traceability, such as stable isotopic ratios, near‐infrared technique, and volatile component. Among them, the multielement method was time‐saving because all the elements can be determined once for each sample. In addition, the concentration of elements in samples was very stable compared with organic composition since some organic compositions in food may change with the temperature, sunshine, and storage time. As a result, multielement analysis was a promising method for mung bean geographical traceability and is more suitable for application in marketing. However, the origin characteristic elements have many factors affected by the method, such as variety, year, soil, moisture, agricultural management measures, sample tracing scope, and sample quantity. Therefore, it is necessary to conduct a systematic study on the methods to provide a theoretical basis for the establishment of standardized methods and database of mineral element origin traceability.

## 5 Conclusion

In this study, the origin tracing model was established for mung bean samples from Dorbod Mongol Autonomous and Tailai County in Heilongjiang Province, Baicheng City in Jilin Province, and Sishui County in Shandong Province. The influences of sample tracing scope and sample quantity on the discrimination results of geographical origins of mung beans based on mineral elements were further explored. The mineral element tracing technology can distinguish mung beans from Dorbod Mongol Autonomous and Tailai County in Heilongjiang Province, Baicheng City in Jilin Province, and Sishui County in Shandong Province. The origin tracing model established based on the samples from the larger scale or more samples had higher accuracy and stability. The influences of sample tracing scope and sample quantity on discrimination results were analyzed. It is concluded that the larger scale from the sample origin, the better the discriminant effect. With the increase in the quantity of samples, the discrimination effect was improved gradually. The accuracy rate of the origin traceability model established using a small sample quantity (*n* = 200) of mung beans was 99.0%. The accuracy rate of the origin traceability model established with a larger sample quantity (*n* = 600) of mung beans increased to 99.33%. The accuracy rate of the origin traceability model established based on the small scale (Tailai, Dorbod Mongol Autonomous, and Baicheng) was 99.55%. The accuracy rate of the origin traceability model established with the larger scale (Northeast region and Shandong Province) increased to 100.0%. This paper compared and analyzed the traceability scope and the quantity of samples from the four geographical origins in China, and the larger tracing scope and the more diverse quantity of samples have not been selected to study the discrimination effect. In future studies, the sample tracing scope and sample quantity can be further expanded to improve the accuracy and stability of the model, give a method for the next step to establish the product database, and provide a useful supplement for the method standardization and application of this method in the future.

## Author Contributions

**Mingming Chen:** writing – original draft (equal), writing – review and editing (equal). **Zhigang Quan:** writing – original draft (equal), writing – review and editing (equal). **Lili Qian:** conceptualization (equal), funding acquisition (equal). **Dongjie Zhang:** conceptualization (equal), visualization (equal).

## Funding

The Heilongjiang Province Mixed Cereals Industry Technology Collaborative Innovation System Mixed Cereals Quality Traceability Technology Post, Heilongjiang Province Characteristic Discipline Funding Project (Heilongjiang Education Union [2018] No. 4).

## Conflicts of Interest

The authors declare no conflicts of interest.

## Data Availability

Data is contained within the article or Supporting Information.
